# Functional Diversity of Novel Lectins with Unique Structural Features in Marine Animals

**DOI:** 10.3390/cells12141814

**Published:** 2023-07-09

**Authors:** Tomomitsu Hatakeyama, Hideaki Unno

**Affiliations:** 1 Biomolecular Chemistry Laboratory, Graduate School of Engineering, Nagasaki University, Bunkyo-machi 1-14, Nagasaki 852-8521, Japan; unno@nagasaki-u.ac.jp; 2 Organization for Marine Science and Technology, Nagasaki University, Bunkyo-machi 1-14, Nagasaki 852-8521, Japan

**Keywords:** lectin, carbohydrate, marine animal, toxin, pore-forming protein, innate immunity

## Abstract

Due to their remarkable structural diversity, glycans play important roles as recognition molecules on cell surfaces of living organisms. Carbohydrates exist in numerous isomeric forms and can adopt diverse structures through various branching patterns. Despite their relatively small molecular weights, they exhibit extensive structural diversity. On the other hand, lectins, also known as carbohydrate-binding proteins, not only recognize and bind to the diverse structures of glycans but also induce various biological reactions based on structural differences. Initially discovered as hemagglutinins in plant seeds, lectins have been found to play significant roles in cell recognition processes in higher vertebrates. However, our understanding of lectins in marine animals, particularly marine invertebrates, remains limited. Recent studies have revealed that marine animals possess novel lectins with unique structures and glycan recognition mechanisms not observed in known lectins. Of particular interest is their role as pattern recognition molecules in the innate immune system, where they recognize the glycan structures of pathogens. Furthermore, lectins serve as toxins for self-defense against foreign enemies. Recent discoveries have identified various pore-forming proteins containing lectin domains in fish venoms and skins. These proteins utilize lectin domains to bind target cells, triggering oligomerization and pore formation in the cell membrane. These findings have spurred research into the new functions of lectins and lectin domains. In this review, we present recent findings on the diverse structures and functions of lectins in marine animals.

## 1. Introduction

Lectins are carbohydrate-binding proteins that play significant roles in the interactions between biological molecules and cells by recognizing specific carbohydrate chains. They are present in a wide range of organisms, from viruses to higher mammals, and function in the recognition of complex glycan structures of great variety due to the combinations of constituent monosaccharides and their linkages [1,2]. Originally discovered in plant seeds, lectins agglutinate erythrocytes by binding to cell surface glycans and crosslinking them. They play crucial roles in recognizing cell surface glycans as glycoproteins and glycolipids, which are essential for maintaining the cell membrane structure and function as receptors to transmit intercellular information. Lectins have also been found in various animal tissues and fluids and serve as essential tools in the recognition of molecules and cells, especially in cell adhesion, immunity, and molecular transport [3].

Animal lectins are categorized into several families based on the structures of their carbohydrate-recognition domains (CRDs) [3,4]. Among them, galectins [5] and C-type lectins [6] are the two major families, which are widely distributed in various organs and tissues and include several subfamilies. Galectins are characterized by their binding specificity for β-galactosides, such as N-acetyllactosamine (Galβ1-4GlcNAc) [5,7]. The CRD of galectins shares a common structural feature of a β-sandwich composed of approximately 135 amino acid residues. Galectins have a wide range of functions, including development/differentiation, apoptosis, cell adhesion, and RNA splicing. In contrast to galectins, whose specificities are mostly restricted to β-galactosides, the C-type lectins, named based on their Ca^2+^-dependency [8], contain CRDs that vary in carbohydrate-binding specificities. In mammals, most of the C-type CRDs are found as carbohydrate-binding modules fused to different domains [6]. One Ca^2+^ ion is usually located in its carbohydrate-binding site and serves to bind specific carbohydrates by forming coordinate bonds [9]. A number of C-type CRD-containing receptors play important roles in the innate immune system [10,11,12].

Other families of animal lectins also play crucial roles in various cellular functions, such as protein folding (calnexin, calreticulin) [13,14], transport of secretory proteins (L-type lectin) [15], transport of lysosomal proteins (P-type lectin) [16], and cell adhesion and immune responses (siglec, I-type lectin) [17,18,19]. These lectins are localized to more specific sites to perform specialized functions involving the recognition of carbohydrate chains. The involvement of various lectins in the recognition processes between intercellular molecules indicates the importance of carbohydrate chains as versatile tags that distinguish molecules.

While most information concerning the structure and function of animal lectins has been obtained from higher vertebrates, particularly mammals, an increasing number of studies on lectins of other species, including invertebrates, have been reported recently, showing that they play important roles in their defense mechanisms. Since invertebrates lack acquired immunity, lectins may be particularly important in innate immunity by recognizing foreign molecules. In particular, various invertebrate C-type lectins have been found to recognize foreign microorganisms and viruses as pattern-recognition molecules [20]. These lectins bind relatively simple carbohydrates with multiple binding sites, thereby recognizing characteristic molecular patterns involving carbohydrates on their surfaces. In addition to C-type lectins that have been shown to be closely associated with the immune system, galectins have recently been revealed to be involved in innate immunity as pattern recognition molecules or effector factors that prevent infection against pathogens [21,22,23,24].

This review will mainly focus on lectins possessing unique structural features from marine animals, particularly invertebrates. These animals encompass an extremely diverse group of species, and therefore, novel lectins with unique structures and functions are expected to exist. Elucidating their structures and functions may provide valuable insights into the roles of lectins from an evolutionary perspective.

## 2. Involvement of C-Type Lectins in Immunity

C-type lectins contain C-type CRDs comprising 120–130 amino acid residues, which require Ca^2+^ ions for their carbohydrate-binding activity. However, various proteins with C-type CRD-like structures that function without Ca^2+^ have also been found, and they are referred to as C-type lectin-like domains (CTLDs). Proteins containing CTLD play a wide range of functions and can recognize ligands other than carbohydrates, such as proteins, lipids, and inorganic compounds [6,9]. Many C-type lectins are known to be involved in innate immunity as pattern recognition molecules [25]. In mammals, most C-type lectins are composed of C-type CRDs and different domains to exert specific functions. Particularly, many C-type lectins are involved in immunity in both soluble and membrane-bound forms. The latter includes a variety of receptors expressed on the surface of immune cells through the transmembrane domain [11].

The mammalian mannose-binding lectin (MBL) was the first C-type lectin to have its CRD structure determined by X-ray crystallography [26]. MBL is a soluble C-type lectin found in mammalian serum, composed of an N-terminal collagen-like domain and a C-type CRD. It forms a trimer linked at its collagen-like and cysteine-rich domains, which further assemble into larger bouquet-like oligomers. In this structure, the carbohydrate-binding sites of the CRDs are oriented in the same direction to bind to the surface mannose-rich glycans on microorganisms. MBL acts as a pattern recognition molecule that recognizes characteristic structures of microorganisms composed of clusters of mannose, thereby activating the lectin pathway of the complement system through the activation of MBL-associated serine proteases (MASPs) [27,28,29]. Similar C-type lectins that contain collagen-like domains are categorized as collectins, including the pulmonary surfactant proteins SP-A and SP-D [30]. Membrane-bound C-type lectins on the surface of lymphocytes, macrophages, and dendritic cells serve as receptors for the surface molecules of pathogens [31].

In contrast to the C-type CRDs found in mammals, many invertebrate C-type lectins consist solely of a single C-type CRD in a polypeptide chain, while some are composed of multiple CRDs or other functional domains [32,33,34]. Invertebrate C-type lectins have been shown to agglutinate microorganisms by binding to surface molecules such as lipopolysaccharides, peptidoglycans, and β-glucans, which leads to phagocytosis [33,35,36,37,38,39,40]. Additionally, their expression is enhanced when challenged with microorganisms [33,37,40,41]. These observations strongly suggest that invertebrate C-type lectins function as pattern-recognition molecules and play a role in innate immunity. The importance of C-type lectins for the immune system in invertebrates is supported by the wide distribution of C-type CRDs or CTLDs among different animal species [42,43,44].

## 3. Carbohydrate Recognition Mechanisms of Marine Invertebrate C-Type Lectins

**Figure 1:**
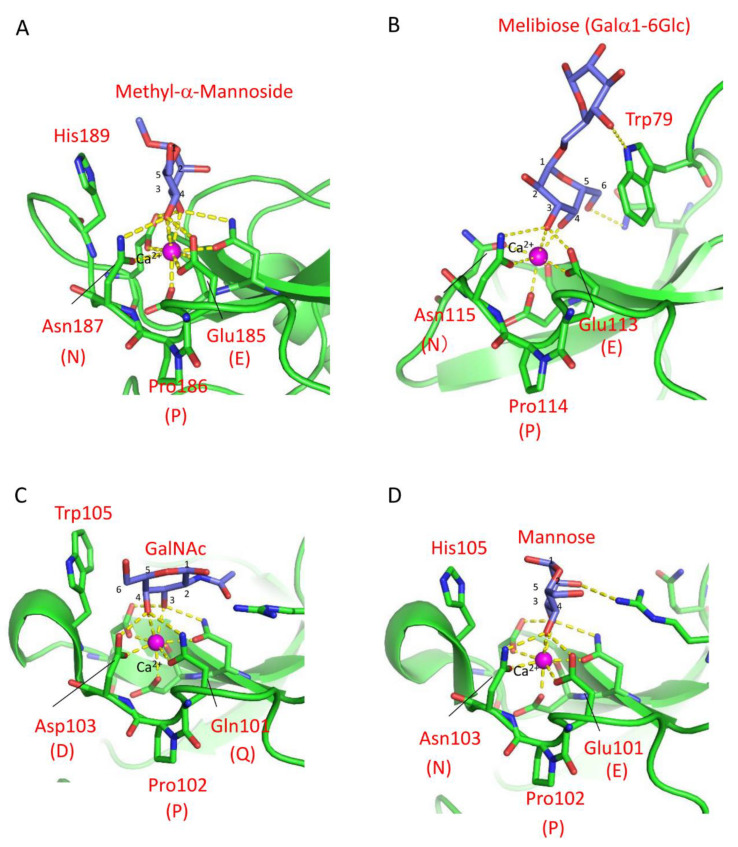
Carbohydrate-binding site structures of the C-type lectins. Coordinate bonds and hydrogen bonds are depicted by yellow dashed lines. (**A**) Mannose-binding lectin (MBP-A)/methyl α-mannoside complex (PDB ID: 1KWU). The orientation of the 4-hydroxy group is determined by hydrogen bonds with the EPN motif. In addition, a histidine residue (His189) stabilizes the binding through stacking interaction. (**B**) CEL-IV/melibiose complex (PDB ID: 3ALT). Despite the presence of the EPN motif, the binding of galactose is stabilized through a stacking interaction between the apolar face of galactose and a tryptophan residue (Trp79). (**C**) CEL-I/GalNAc complex (PDB ID: 1WMZ). The binding of GalNAc is stabilized by Trp105 through a hydrophobic interaction. (**D**) The mannose-binding mutant EPNH-CEL-I/mannose complex (PDB ID: 4WQQ). Mannose is recognized by the EPN motif and further stabilized by a histidine residue (His105).

The carbohydrate-binding mechanism of C-type lectin CRDs has been elucidated through X-ray crystallographic analysis of rat mannose-binding lectin (MBL) MBP-A [26]. The CRD of MBP-A contains three Ca^2+^ ions at the top of the domain, one of which is involved in mannose-binding through coordinate bonds with the 3- and 4-hydroxy groups of mannose (Figure 1A) [6]. The binding of mannose is further stabilized by a hydrogen bond network between the 3- and 4-hydroxy groups and nearby residues, Glu185 and Asn187. In addition to Pro186, these residues are known as the EPN (Glu-Pro-Asn) motif that determines the mannose specificity of C-type CRDs, while the QPD (Gln-Pro-Asp) motif is associated with galactose specificity [45]. These three-amino-acid motifs differ in the hydrogen donor/acceptor pair at the first and third positions (Glu/Gln and Asn/Asp), leading to a difference in the orientations of the hydroxy groups at the 3- and 4-positions of the bound monosaccharides, discriminating between mannose and galactose.

Although the relationship between mannose/galactose specificity and the motifs applies to many C-type CRDs, different motifs have been found in invertebrate C-type CRDs [32,46,47,48,49], suggesting the diversity of binding specificity in these lectins. For example, despite having the EPN motif (Glu113-Pro114-Asn115) in the binding site, the C-type lectin CEL-IV from sea cucumber (*Cucumaria echinata*) recognizes galactose [50]. X-ray crystallographic analysis of CEL-IV/carbohydrate complexes revealed that the nonreducing galactose residue of melibiose was recognized in an inverted orientation compared to methyl α-mannoside bound to MBP-A containing the EPN motif in terms of their 3- and 4-hydroxy groups, as shown in Figure 1A,B. This recognition mode was facilitated by a stacking interaction with a tryptophan residue (Trp79) (Figure 1B). A similar contribution of tryptophan residues to the recognition of galactose was also exemplified [51]. The significance of stacking interactions between the galactose residue and aromatic side chains was further demonstrated in the mutant of CEL-I, another *C. echinata* C-type lectin [52,53]. Although mutations from the QPD to EPN motifs were insufficient to change its binding preference from N-acetylgalactosamine to mannose, the additional mutation of a tryptophan residue (Trp105) that forms the stacking interaction with the hydrophobic face of galactose to histidine resulted in a higher affinity for mannose than GalNAc (Figure 1C,D).

While most invertebrate C-type lectins are known to be Ca^2+^-dependent, there are some lectins that exhibit Ca^2+^-independent binding activity [47,54,55]. For instance, the bivalve *Saxidomus purpuratus* possesses two isolectins, SPL-1 and SPL-2, showing specificity for GlcNAc and GalNAc [54], although typical C-type CRDs exhibit distinct specificities for these monosaccharides, which have different configurations of the 4-hydroxy groups. The crystal structure of the SPL-2/GalNAc complex revealed that the bound GalNAc is primarily recognized through stacking interactions with tyrosine and histidine residues, as well as hydrogen bonds with aspartate and asparagine residues. In fact, the QPD/EPN motifs are replaced by RPD (Arg-Pro-Asp) or KPD (Lys-Pro-Asp) in the subunits of SPL-1 and SPL-2, which are not directly involved in carbohydrate recognition. While Ca^2+^ is not essential for carbohydrate binding, its presence can enhance the binding affinity of SPL-1 and SPL-2 by coordinating two water molecules that form hydrogen bonds with the 3- and 4-hydroxy groups of the carbohydrates [56]. These lectins likely function in recognizing the acetamido groups present on the surface molecules of microorganisms. The diverse ligand recognition abilities of CTLDs may be achieved through structural variations in their binding sites, which are supported by the versatile fold of the C-type CRD.

## 4. Marine Animal Lectins with Novel Structures and Carbohydrate-Recognition Mechanisms

### 4.1. Mannose-Binding Lectin CGL1 from the Pacific Oyster

**Figure 2:**
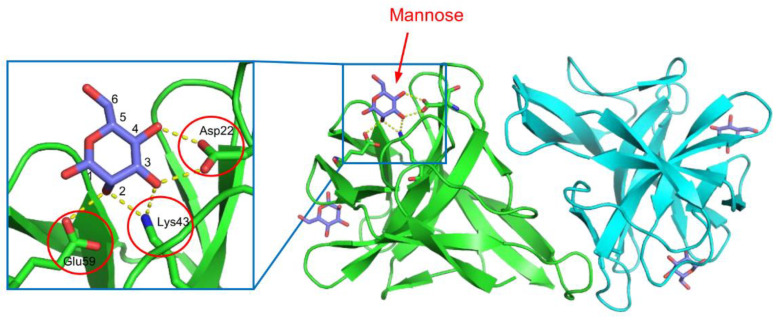
The crystal structure of the CGL1/mannose complex. Two subunits are colored green and cyan. The bound mannose molecules are depicted using a stick model, and hydrogen bonds are indicated by yellow dashed lines. The residues involved in hydrogen bonding with mannose are enclosed by red circles.

CGL1 (CgDM9CP-1) is a mannose-binding lectin isolated from the Pacific oyster (*Crassostrea gigas*) [57,58]. This lectin consists of two identical subunits of 15.5 kDa. The protomer of CGL1 consists of tandemly repeated sequences of approximately 70 amino acid residues, which exhibit homology with DM9 repeats found in the fruit fly (*Drosophila melanogaster*) genome [59]. This lectin possesses a unique fold with the DM9 domain structure, distinct from other known lectins [57]. The two carbohydrate-binding sites are located in the pockets between the two DM9 domains. In the crystal structure of the CGL1/mannose complex (Figure 2), the bound mannose molecules are recognized through hydrogen bonds formed between the 2-, 3-, and 4-hydroxy groups of mannose and the side chains of Asp22, Lys43, and Glu39, resulting in high specificity for terminal mannose in various oligosaccharides. This is consistent with the results obtained from frontal affinity chromatography and glycan array measurements [57,58]. Besides CGL1, similar DM9 domain lectins have been identified in *C. gigas*, and they have been suggested to be involved in innate immunity based on their ability to bind molecules derived from foreign microorganisms, such as lipopolysaccharide, peptidoglycan, mannan, and β-1,3-glucan [58,60,61].

While DM9 domain proteins from *C. gigas* have demonstrated mannose-specific lectin activity, the functions of most DM9 domain proteins from other species remain unclear. For instance, the DM9 protein from the parasitic flatworm (*Fasciola gigantica*) (FgDM9-1) has been shown to possess mannose-binding ability and can agglutinate erythrocytes and bacteria [62]. Interestingly, FgDM9-1 was found to be relocated to vesicular structures within the cell under conditions of bacterial, drug, and heat stresses [63]. A similar intracellular relocation into vesicle-like structures has been observed for another DM9 domain protein, PRS1, from the mosquito (*Anopheles gambiae*) [64]. PRS1 was induced in the epithelial cells of the salivary glands upon invasion by the malaria parasite *Plasmodium*, and it was also relocated to vesicle-like structures. These findings suggest that DM9 proteins may be involved in innate immunity by interacting with mannose-containing molecules and related molecules of pathogens and parasites. In vertebrates, DM9 repeat motifs have only been identified as domains of proteins from fish venoms and skins [65,66].

### 4.2. Galactose-Binding Lectin AJLec from Sea Anemone

**Figure 3:**
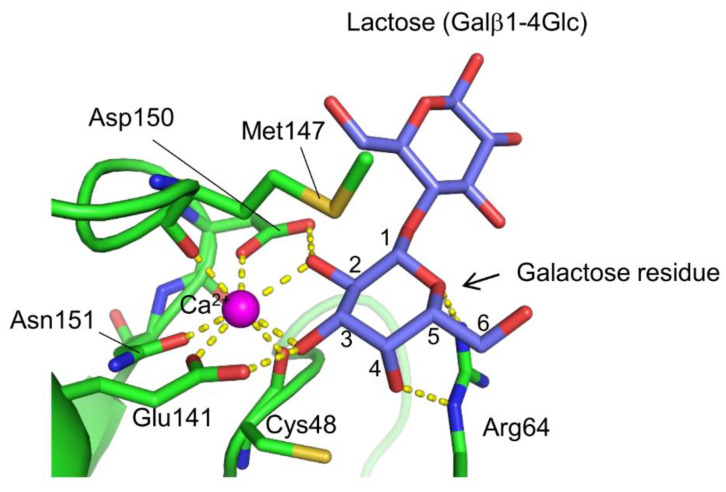
Carbohydrate recognition mode of AJLec (PDB ID: 6A56). The galactose residue of the bound lactose is recognized through coordinate bonds with the Ca^2+^ ion and hydrogen bonds with specific amino acid side chains, resembling the recognition patterns observed in C-type lectins. However, unlike galactose-specific C-type lectins, AJLec is unable to bind N-acetylgalactosamine due to its recognition of the 2-hydroxy group of the galactose residue. The coordinate bonds and hydrogen bonds are indicated by yellow dashed lines.

A novel lectin named AJLec (18.5 kDa) was discovered in the cnidarian *Anthopleura japonica* (sea anemone) [67]. AJLec is a dimeric lectin composed of two identical subunits linked by two disulfide bonds, forming a helical-rod shape with lactose binding sites located at both termini. AJLec exhibits Ca^2+^-dependent carbohydrate-binding activity, similar to C-type lectins. However, its amino acid sequence and tertiary structure show no resemblance to any known lectins, including C-type lectins. In the carbohydrate-binding site (Figure 3), one Ca^2+^ ion is located, forming two coordinate bonds with the 2- and 3-hydroxy groups of the galactose residue of lactose. Binding is further stabilized by hydrogen bonds with nearby residues, including Ca^2+^-coordinating ones (Glu141 and Asp150). Arg64 appears crucial for recognizing galactose as it forms a hydrogen bond with the 4-axial hydroxy group of the galactose residue. This represents a significant difference in the galactose recognition mode compared to galactose-specific C-type CRDs, which form coordinate bonds and hydrogen bond networks between Ca^2+^ and the galactose 3- and 4-hydroxy groups. AJLec preferentially binds to galactose and glycoproteins with β-linked terminal galactose residues but not to N-acetylgalactosamine (GalNAc), which is commonly recognized by many other galactose-binding lectins. This difference is due to the involvement of a coordinate bond between the Ca^2+^ ion and the 2-hydroxy group of galactose, which is replaced by an acetamido group in N-acetylgalactosamine.

Notably, genes encoding AJLec homologues are exclusively found in cnidarians, such as sea anemones and corals. This may be related to unique features in the life of these organisms. On the other hand, the dimeric structure of AJLec with carbohydrate-binding sites protruding on both sides of the molecule suggests a potential role in crosslinking carbohydrate chains on invading microorganisms, leading to their neutralization or activation of phagocytes.

## 5. Lectins as Toxins from Marine Animals

Many plant lectins have the ability to induce cellular toxicity, likely due to their role in self-defense against invading organisms such as pathogenic bacteria or insects [68,69]. This toxicity arises from their capacity to cross-link surface carbohydrate-containing molecules on target cells, influencing the signaling pathways within those cells. Moreover, when incorporated into protein toxins, lectin domains can function as cell-binding modules to exert potent toxicity. A well-known example of a toxic lectin is ricin, which was the first discovered toxic lectin derived from the castor bean (*Ricinus communis*) [70]. Ricin consists of an A chain with N-glycosidase activity that inactivates eukaryotic ribosomes and a B chain that serves as a lectin subunit. The B chain assists in delivering the A chain to the cytosol by binding to cell surface glycans [71,72]. Similar toxins with enzymatic “A” subunits and receptor-binding “B” subunits (lectin subunits) are referred to as AB toxins and are also produced by various pathogenic bacteria [73]. Another type of toxic lectin includes pore-forming proteins, which form ion-permeable pores in the target cell membrane after binding to cell surface glycans through the lectin domains. These examples demonstrate the important roles that lectins often play in the actions of protein toxins. It has also been revealed that venomous marine animals produce various toxins containing lectin domains, suggesting a close association between their carbohydrate-binding activity and their toxic actions.

### 5.1. Lectin from the Venomous Sea Urchin Toxopneustes pileolus

**Figure 4:**
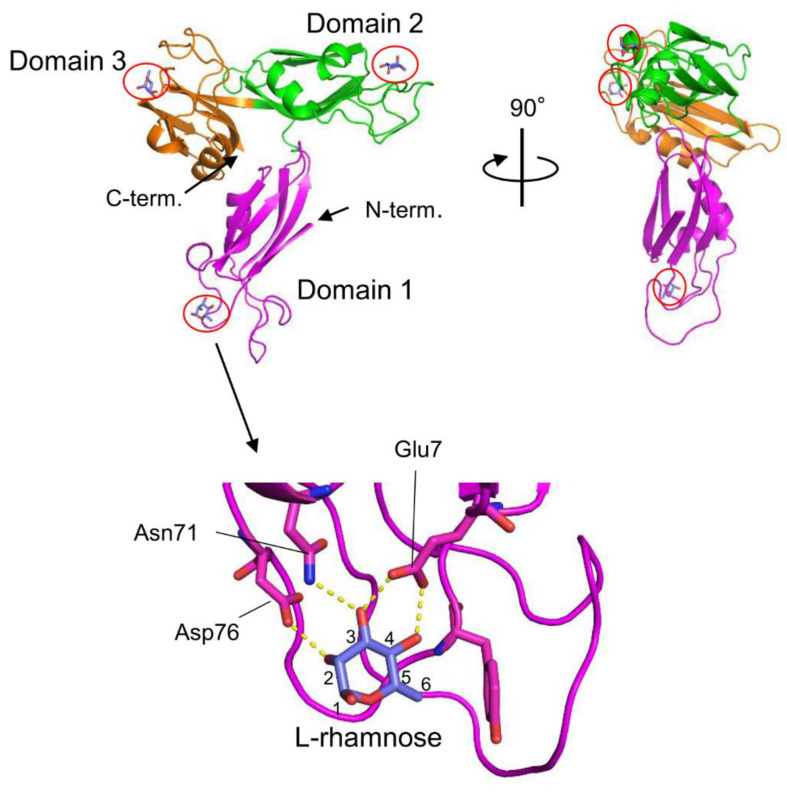
Crystal structures of SUL-I complexed with L-rhamnose (PDB ID: 5H4S). SUL-I is composed of three distinct domains (domains 1–3), each possessing a carbohydrate-binding site at its apex. The domains are presented in different colors. The bound L-rhamnose molecules are enclosed by red circles. In domain 1, L-rhamnose is recognized through hydrogen bonds formed between its 2-, 3-, and 4-hydroxy groups and Asp76, Asn71, and Glu7. L-Rhamnose molecules are similarly recognized in domains 2 and 3.

The venom of the globiferous pedicellariae of the venomous sea urchin *T. pileolus* contains several toxic proteins that exert various biological effects [74]. Among these proteins, the galactose-specific lectin SUL-I exhibits various activities, such as chemotactic activity on guinea pig neutrophils and mitogenic activity on murine splenocytes [75,76]. The amino acid sequence of SUL-I indicates that it belongs to the L-rhamnose-binding lectins (RBLs), many of which have been found in fish eggs [77]. SUL-I is composed of 284 residues (30.5 kDa) with three repetitive sequence regions, each showing similarity to CRDs of rhamnose-binding lectins (RBLs) [78]. The three-dimensional structure of the rSUL-I/L-rhamnose complex was determined by X-ray crystallographic analysis with a resolution of 1.8 Å (Figure 4) [79]. The overall structure of rSUL-I consists of three distinctive CRDs, which share structural similarities with the CRDs of CSL3, the rhamnose-binding lectin from chum salmon (*Oncorhynchus keta*) eggs [80]. The bound L-rhamnose molecules are primarily recognized by rSUL-I through hydrogen bonds between their 2-, 3-, and 4-hydroxy groups and Asp, Asn, and Glu residues in the binding sites. When interactions of rSUL-I with oligosaccharides were examined, a higher affinity was observed for galactosylated (asialylated) N-glycans, suggesting that the potential target carbohydrate structures are galactose-terminated N-glycans. While the CRDs of rSUL-I adopt similar folds compared to those of CSL3, a significant difference was found around the variable loop in the carbohydrate-binding sites, which may be related to the difference in binding specificities for oligosaccharides between these lectins. SUL-I has carbohydrate-binding sites in its three domains, all oriented towards the same side of the protein. This structure could be advantageous for cross-linking specific membrane glycoproteins containing galactose-terminated carbohydrate chains, thereby triggering cellular responses.

### 5.2. Hemolytic Lectin from the Sea Cucumber C. echinata

**Figure 5:**
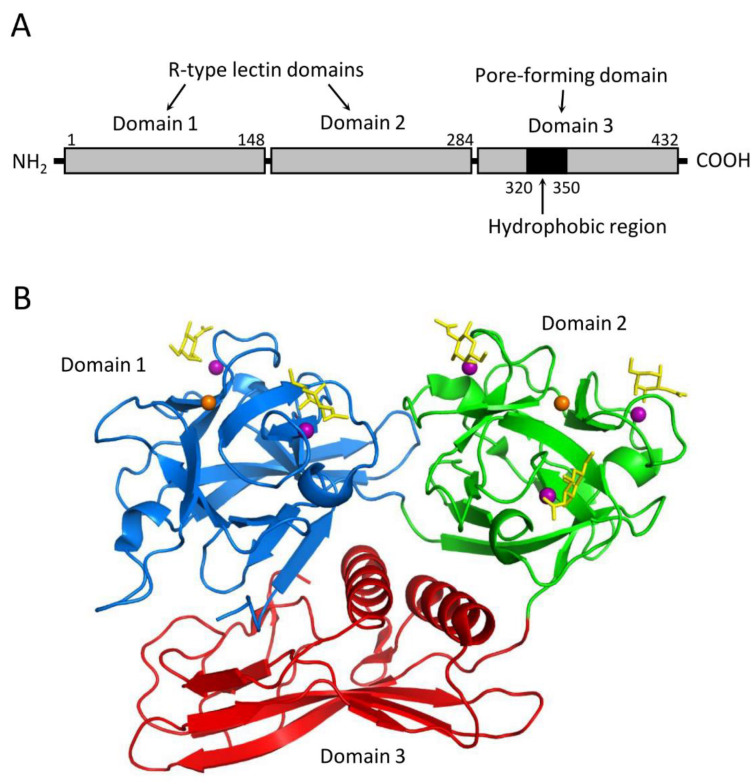
Structure of the hemolytic lectin CEL-III (PDB ID: 2Z48). (**A**) CEL-III comprises three domains. Domains 1 and 2 are R-type (β-trefoil) lectin domains, while domain 3 is a pore-forming domain that contains a hydrophobic region. (**B**) Crystal structure of the CEL-III/GalNAc complex. Domains 1 and 2 bind galactose-related carbohydrates by utilizing Ca^2+^ ions in a similar manner to C-type lectins. Domain 3 exhibits a unique structure abundant in β-strands, along with two α-helices that correspond to the hydrophobic region shown in (**A**). The bound GalNAc molecules are depicted as yellow stick models.

CEL-III is a galactose-specific lectin from *C. echinata*, which also contains the previously mentioned C-type lectins CEL-I and CEL-IV. Although CEL-III shows Ca^2+^-dependent carbohydrate binding, it belongs to the R-(ricin)-type lectin family, characterized by two β-trefoil folds [81]. Interestingly, it exhibits strong hemolytic activity and cytotoxicity [82]. Hemolysis induced by this lectin is prominent in the alkaline region above pH 7 in the presence of Ca^2+^ and can be inhibited by galactose and carbohydrates containing galactose at nonreducing ends. These observations suggest that the Ca^2+^-dependent galactose binding ability is a prerequisite for its hemolytic action. CEL-III mediates hemolysis by forming pores in the target erythrocytes, relying on its galactose-specific lectin activity. Structural analyses of CEL-III have revealed that it consists of two R-type lectin domains (domains 1 and 2) in the N-terminal two-thirds and a C-terminal one-third with a β-sheet-rich novel domain (domain 3) (Figure 5) [83,84]. Domain 1 contains two carbohydrate-binding sites, while domain 2 contains three. The binding of galactose-related carbohydrates is mediated by coordinate bonds with Ca^2+^ ions bound in the carbohydrate-binding sites, along with hydrogen bond networks involving nearby amino acid residues. This mechanism shares similarities with the carbohydrate-recognition mechanism of C-type CRDs, despite the lack of homology in the amino acid sequences between CEL-III and C-type CRDs.

**Figure 6:**
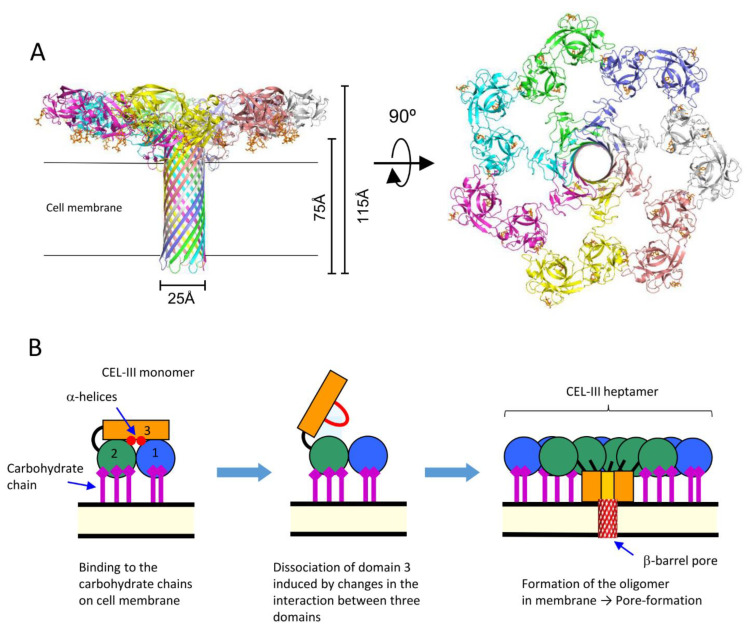
Oligomerization of CEL-III. (**A**) The crystal structure of the CEL-III heptamer (PDB ID: 3W9T). Monomer units of CEL-III are depicted in different colors. The α-helical regions in domain 3 have changed to a β-barrel structure, which is assumed to penetrate the target cell membrane. (**B**) Schematic drawing for presumed oligomerization processes. After binding to carbohydrate chains on the target cell surface, domain 3 may dissociate from domains 1 and 2. Concomitantly with heptamerization, the α-helical regions of domain 3 change their conformation to β-strands, forming a transmembrane pore composed of a β-barrel.

The crystal structure of the soluble oligomer of CEL-III, induced by the binding of lactulose, a galactose-containing disaccharide, revealed that CEL-III heptamerizes upon binding to galactose-containing carbohydrates (Figure 6). This binding leads to the formation of a β-barrel structure (25 Å) composed of the C-terminal β-sheet-rich domain [84]. When these conformational changes occur on the target cell surface, the β-barrel forms a membrane pore, allowing the passage of small molecules and ions across the cell membrane. This drastic change in the tertiary structure of domain 3, from a soluble form to a membrane-penetrating β-barrel, is driven by the formation of numerous hydrogen bonds within the β-barrel and hydrophobic interactions between the hydrophobic face of the β-barrel and the nonpolar interior of the membrane.

Oligomerization of CEL-III can be triggered by β-galactoside-containing disaccharides, including lactose (Galβ1-4Glc), lactulose (Galβ1-4Fru), and N-acetyllactosamine (Galβ1-4GlcNAc), but not by galactose, N-acetylgalactosamine, and melibiose (Galα1-6Glc) [85]. This suggests that heptamerization of CEL-III on the target cell surface is induced by cell surface receptors containing β-galactoside linkages, particularly glycosphingolipids such as lactosyl ceramide, which has been demonstrated to be an effective receptor for membrane pore formation in artificial lipid vesicles [86]. It appears that the drastic conformational change of CEL-III is triggered by the structural changes in the binding of the specific carbohydrate into domains 1 and 2, which are then transmitted to domain 3, leading to the opening of the interface with domain 3 [84].

The presence of β-trefoil fold domains containing QXW motifs [87] has been observed in a wide range of proteins, including lectins, toxins, carbohydrate-related enzymes, and immune cell surface receptors, which function as carbohydrate-binding modules. Structural analysis of the hemolytic lectin LSL, isolated from the mushroom *Laetiporus sulphureus*, has revealed that it also comprises an N-terminal carbohydrate-binding domain with a β-trefoil fold and a C-terminal pore-forming domain [88]. Notably, the pore-forming domain of this lectin exhibits remarkable structural similarity to those found in pore-forming toxins of pathogenic bacteria such as *Aeromonas hydrophila* and *Clostridium perfringens* [89]. Based on these findings, it can be inferred that LSL, similar to CEL-III, exhibits hemolytic activity by binding to cell surface glycans through its carbohydrate-binding domain and subsequently inserting a pore-forming domain into the cell membrane. Although the pore-forming domain of LSL does not share structural similarity with domain 3 of CEL-III, both domains are characterized by elongated β-sheets, which likely contribute to a favorable structure for penetrating the cell membrane.

### 5.3. Fish Spine Toxins Containing Lectin-like Domains

**Figure 7:**
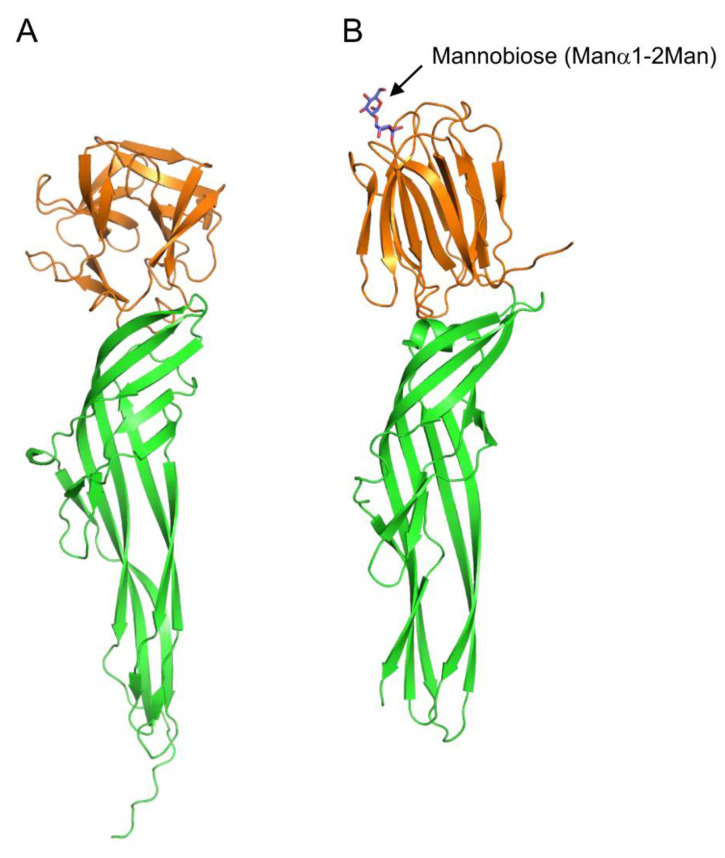
Comparison of tertiary structures of Ij-natterin and Dln1/mannobiose complex. (**A**) The tertiary structure of Ij-natterin predicted using Alphafold2 [93]. The N-terminal 50 residues are omitted due to their disordered nature. (**B**) The X-ray crystal structure of Dln1/mannobiose (Manα1-2Man) complex (PDB ID: 4ZNQ). The N-terminal domain demonstrates mannose-specific lectin activity. The bound mannobiose molecule is depicted as a stick model. In both proteins, the N- and C-terminal domains are colored orange and green, respectively.

Some fish species possess stinging spines that contain protein toxins; among them, a toxin called natterin has been isolated from the spines of *Thalassophryne nattereri* [65]. Additionally, a homologous protein Ij-natterin has been found to be expressed in the spine venom of *Inimicus japonicus* [66]. Natterin and Ij-natterin consist of an N-terminal lectin domain, which is homologous to the oyster lectin CGL1 (DM9 domain protein) [57], and a C-terminal aerolysin-like pore-forming domain (Figure 7A). Although the specific carbohydrate-binding activity of the lectin domain has not been reported to date, its amino acid sequence similarity with CGL1 suggests that it may have mannose-specific lectin activity.

Proteins that have C-terminal domains homologous to those of natterin, called natterin-like proteins, are also widely distributed in fish skin [90,91]. The crystal structure of Dln1, one of the natterin-like proteins from zebrafish (*Danio rerio*), has revealed that Dln1 consists of an N-terminal jacalin-like lectin domain and a C-terminal aerolysin-like domain (Figure 7B) [92]. The lectin domain was found to bind mannobiose (Manα1-2Man and Manα1-3Man), indicating that Dln1 exhibits an affinity for high-mannose glycans. Furthermore, electron microscopic analysis revealed that upon binding to high-mannose glycans, Dln1 undergoes significant conformational changes, leading to the formation of octameric pores in the lipid membrane.

Although natterin and natterin-like proteins possess C-terminal aerolysin-like domains in common, they differ in N-terminal lectin domains; the former shows a CGL1-like DM9 domain fold, while the latter has a jacalin-like domain. Considering the large conformational change during the pore-forming process of Dln1, it seems highly likely that natterin (and Ij-natterin) also induce significant conformational changes upon binding to target cell surface glycans, leading to oligomerization and pore formation. Similar large conformational changes after binding to cell surface carbohydrate chains are also observed in the course of pore formation by the hemolytic lectin CEL-III (Figure 6). The lectin domains of these pore-forming proteins not only play roles in binding to specific carbohydrate chains on the target cell but also trigger subsequent conformational changes leading to the formation of oligomers with transmembrane pores.

## 6. Conclusions

Lectins, initially discovered as hemagglutinins in plant seeds, have been found to be widely distributed in living organisms and play crucial roles in molecular and cellular recognition through carbohydrate chains. In this review, our primary focus has been on lectins from marine animals, which exhibit novel structures and functions. Marine animals, especially invertebrates, demonstrate a higher degree of diversity compared to higher vertebrates, as exemplified by the lectins with novel protein folds, such as CGL1, AJLec, CEL-III, natterin, and natterin-like proteins. Therefore, exploring novel proteins with unknown structures and functions among these organisms, including lectins, is expected to yield valuable information. An increasing number of studies have highlighted the crucial roles of lectins in immune systems. Lectins are particularly important in innate immunity as pattern recognition molecules, enabling the recognition of foreign substances. Since information on the innate immune systems of marine invertebrates has been limited thus far, investigating lectins involved in the innate immunity of these animals is highly likely to provide valuable insights into the innate immune systems of marine animals.

Additionally, lectins that act as binding modules for various protein toxins are intriguing targets for understanding biological defense systems as well as the mechanisms of protein-carbohydrate and protein-lipid membrane interactions. Numerous pore-forming proteins containing lectin domains, including those found in marine animals, have been identified. In these pore-forming proteins, lectin domains not only serve as binding modules to target specific cells but also trigger subsequent conformational changes that lead to oligomerization and the formation of membrane-penetrating pore structures. In addition to their role as toxins, pore-forming proteins are involved in various functions in organisms, such as immune systems [94] and apoptosis [95]. Examining the structural changes of pore-forming lectins during the oligomerization process may provide important clues for understanding the conformational changes triggered by receptor binding.

## Data Availability

Not applicable.
